# Organic walled microfossils in wet peperites from the early Cretaceous Paraná-Etendeka volcanism of Brazil

**DOI:** 10.1038/s41598-023-42483-6

**Published:** 2023-09-16

**Authors:** Lucas Del Mouro, Bruno Becker-Kerber, Valdecir A. Janasi, Marcelo de Araújo Carvalho, Breno L. Waichel, Evandro F. Lima, Lucas M. M. Rossetti, Vinicius Cruz, Mateus Souza Silva, Natália Famelli, Javier Ortega-Hernández

**Affiliations:** 1 Department of Organismic and Evolutionary Biology and Museum of Comparative Zoology, Harvard University, Cambridge, MA 02138 USA; 2 Institute of Geosciences, University of São Paulo, São Paulo, Brazil; 3 National Museum/Federal University of Rio de Janeiro, Rio de Janeiro, Brazil; 4 Espepetro, Federal University of Santa Catarina, Florianópolis, Brazil; 5 Federal University of Rio Grande Do Sul, Porto Alegre, Brazil; 6 Federal University of Mato Grosso, Cuiabá, Brazil; 7 CODES - Centre for Ore Deposits and Earth Sciences, University of Tasmania, Hobart, 7001 Australia; 8 Centro de Pesquisas e Desenvolvimento Leopoldo Américo Miguez de Mello – CENPES/PETROBRAS, Rio de Janeiro, Brazil

**Keywords:** Palaeoecology, Systems biology, Palaeontology, Volcanology

## Abstract

Large igneous provinces (LIPs) are major magmatic events that have a significant impact on the global environment and the biosphere, for example as triggers of mass extinctions. LIPs provide an excellent sedimentological and geochemical record of short but intense periods of geological activity in the past, but their contribution towards understanding ancient life is much more restricted due to the destructive nature of their igneous origin. Here, we provide the first paleontological evidence for organic walled microfossils extracted from wet peperites from the Early Cretaceous Paraná-Etendeka intertrappean deposits of the Paraná basin in Brazil. Wet peperites are a volcaniclastic rock formed by the interaction of lava and subaqueous sediments.The Paraná-Etendeka was formed during the Valanginian (ca. 132 Ma) as a continental flood basalt in present day South America and Namibia, and released enormous amounts of carbon dioxide, sulfur dioxide, methane and hydrogen fluoride into the atmosphere. The organic walled microfossils recovered from the Paraná-Etendeka peperites include pollen grains, spores, acritarchs, and other remains of unidentifiable organic matter. In addition to the peperites, organic walled microfossils were also found in heterolithic sandstones and interpillow sandstones. Our findings represent the first insight into the biodiversity of the Paraná Basin during the Early Cretaceous during a period of intense magmatism, and the microfossil assemblages corroborate a regional paleoclimatic transition from arid to more humid conditions that were likely induced by the volcanic activity. We corroborate the potential of wet peperite rocks as a valuable source of paleobiological data and emphasize the importance of sampling volcaniclastic units that have been traditionally considered with lower fossiliferous potential due to their igneous origin.

## Introduction

The Paraná-Etendeka Large Igneous Province (PE-LIP) is a well-known magmatic event^1–8^ from the Early Cretaceous. This volcanic activity is related with the opening of the South Atlantic Ocean, and thought to have contributed to the Weissert Anoxic Event (WE)^9–14^, and to the proliferation of more humid climate conditions in Paraná Basin of Brazil during the Valanginian (c. 140–133 Ma)^15,16^. In particular, the WE is marked by a positive δ^13^C excursion, and is often linked to a minor biotic crisis^13–18^. Although the correlation between the PE-LIP and the WE^13,17,18^ remains uncertain, it has been suggested that CO_2_ degassing processes triggered global warming and affected the hydrological cycle^10,15,18^. Alternatively, it has also been proposed that the PE-LIP might have caused a cooling phenomenon at the end of the volcanism^19–21^. Recent data from sedimentary and volcaniclastic rocks interbedded with PE-LIP lavas in Brazil suggests that the SO_2_ degassing process from volcanism caused a climate cooling and restructured the precipitation pattern^8^, resulting in more humid conditions at the Paraná Basin. Despite the availability of sedimentological and geochemical data, the PE-LIP lavas have not been investigated in terms of their paleobiological significance because igneous rocks are typically considered as non-conducive to the preservation of fossilized biological remains.

In this study, we report an assemblage of organic walled microfossils extracted from wet peperite rocks, sandstones and interpillow sandstones from the PE-LIP in Brazil. Although there have been few mentions of ostracods in this province before^22^, these organic walled microfossils represent the first documented fossil recovered from this LIP. They are critically the first case of paleontological remains known from Mesozoic wet peperite deposits, as previous findings have exclusively recorded at the Palaeogene Staffa Formation^23^.

Unexpectedly, the studied peperite deposits yielded a higher microfossiliferous content in terms of diversity and abundance relative to the volcanoclastic sandstones. This suggests that specific physical–chemical processes related to the peperite formation have contribute to improved preservation of microfossils.

## Materials and methods

### Stratigraphy and palaeoenvironment

**Figure 1:**
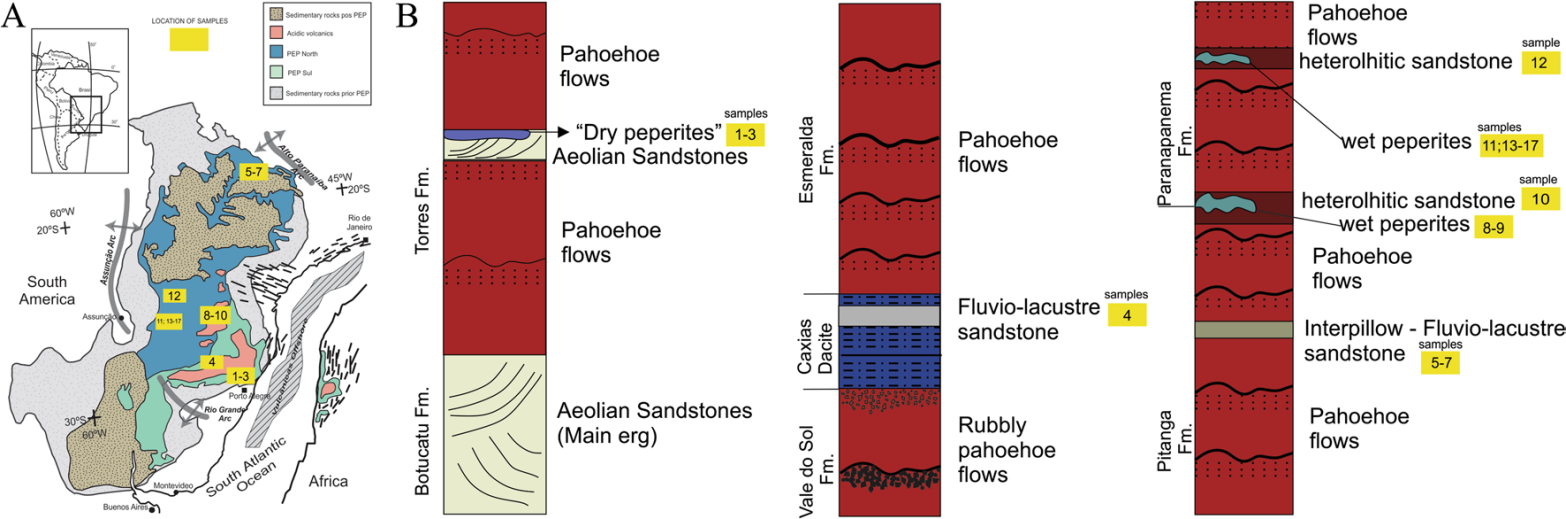
Locality and stratigraphy. (**A**) Locality of sedimentary and volcaniclastics sites in Brazil and Africa. These sites (yellow boxes) are related to Torres, Esmeralda, Pitanga and Paranapanema formations. (**B**) Stratigraphy of the Serra Geral Group. The stratigraphic sequence of the SGG is depicted from the base to the top. It includes the Torres Formation, followed by the Vale do Sol Formation (not evaluated in this study), the Esmeralda Formation,the Pitanga Formation, and the Paranapanema Formation. Outcrop numbers are provided to indicate the number of fertile sites identified within each formation. These fertile sites are distributed across various regions in Brazil. Outcrop numbers: 1—VN26; 2—VEC30A; 3—VEC12; 4—VN12; 5—NFUB03; 6—UBNF32A; 7—UBNF32B (sterile); 8—PR06; 9—PR11; 10—PR02; 11—BS18; 12—BS12; 13—BS23; 14—SC03/BS01; 15—BS22; 16—BS24; 17—BS21. Figure created using Corel Drawn Home & Student X7.

The organic walled microfossils were found in 15 different outcrops distributed along the Brazilian states of Minas Gerais, Paraná, Santa Catarina and Rio Grande do Sul (Fig. 1 and Supplementary 1–3). These outcrops include volcaniclastics and sedimentary deposits (heterolhitic sandstones, peperites and interpillow sandstones) which were deposited in Paraná Basin^23^ during the quiescent phases from the volcanic event of PE-LIP, dated as Lower Cretaceous (Valanginian—Hauterivian)^1–8^. The PE-LIP fall within the Serra Geral Group, which based on lava flow morphologies and products of variations in eruptive dynamics, is subdivided into Torres, Vale do Sol, Palmas and Esmeralda formations^5^, in the south and Pitanga and Paranapanema formations in the north^6^. Although most of the samples were collected from the uppermost unit of the Serra Geral Group (Paranapanema Formation), volcaniclastic rocks from Torres, Esmeralda and Pitanga formations were also considered (Fig. 1).

The studied intertraps correspond to heterolithic sandstones (predominantly by fine sand and small amount of silt/mud) with minor volcanic fragments, which suggest depositional conditions related to ephemeral lakes/ponds^8,24,25^ (Supplementary 1A). Pillow lavas were recognized in the northern part of Serra Geral Group^7^, where interpillow sandstones are predominantly composed of altered vitreous fragments in a non-volcanic (e.g. fine sand and silt/mud) matrix and are interpreted as fluvio-lacustrine deposits^6^ (Supplementary 1B). The interpillow and wet peperite deposits, which are confined to the last moments of the volcanism (Pitanga and Paranapanema), were coeval with the lava flows, indicating the end of the quiescent phase.

**Figure 2:**
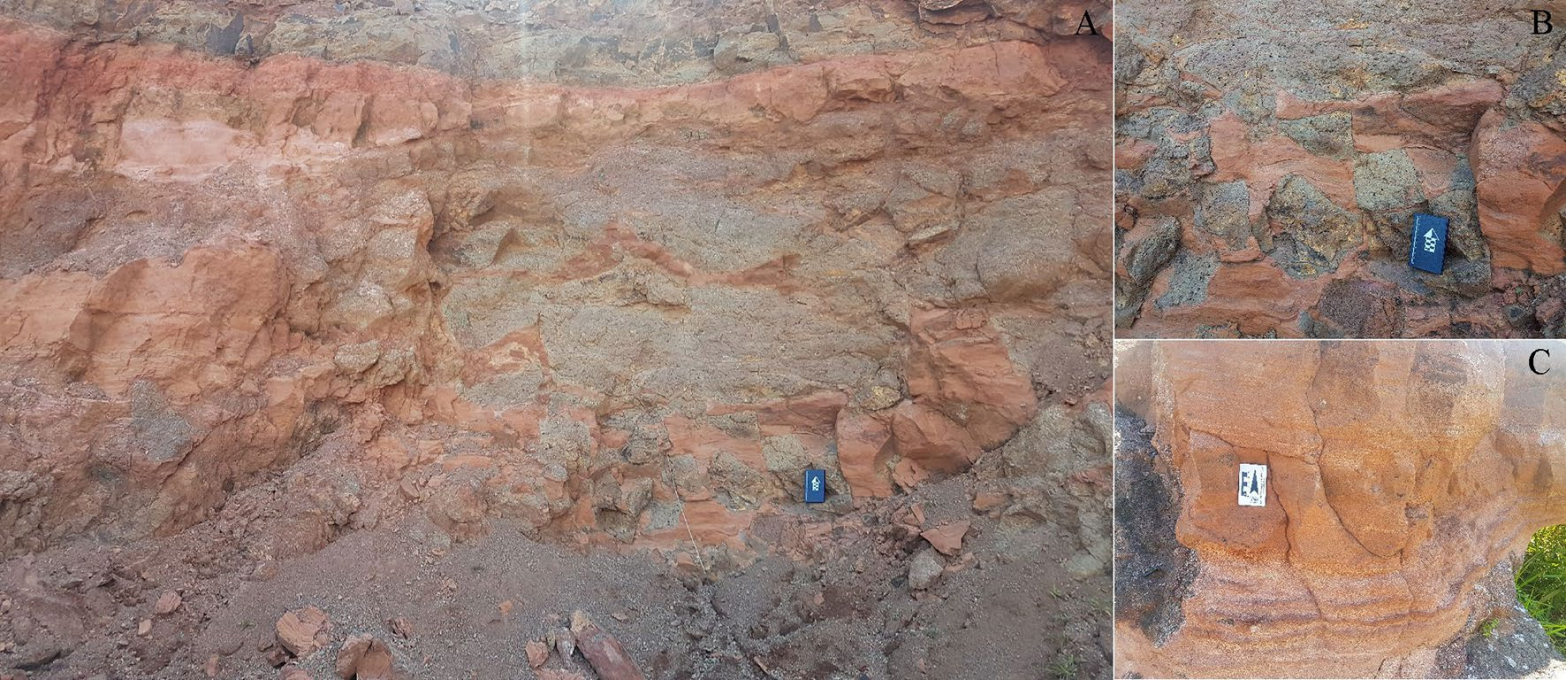
General view of a wet-peperite site located in Paraná Basin. (**A**) An overview of dynamic mixing between the quenched basaltic (?) debris and sediment at SC-03/BS01. (**B**) Detail of the interaction between lava flows and non-volcanic sediments showing soft-sediment deformation and blocky type peperite. (**C**) Thick sandstone with fragments of volcanic rocks.

Peperite rocks are typically formed by the interaction of a lava flow into an unconsolidated sediment, normally under wet conditions^26^. The peperite-forming processes are diverse and result in two categories—dry and wet— both of which are found in Paraná Basin^1–4,9^. These primary volcaniclastic rocks are classified as basaltic breccias set in a non-volcanic matrix with a heterolithic lamination^3,8,25^_._ Dry peperites are formed by lava cascading down from a dune faces under hyper-arid conditions with no evidence of water; within the PE-LIP, dry peperites are restricted to the lowermost Torres Formation (Supplementary 1C)^1,8^. By contrast, wet peperites are formed in subaqueous settings restricted to the upper portion of the volcanic sequence and probably at the border ephemerous subaqueous setting. We identify two types^3^ of wet peperites in the upper formations (Pitanga and Paranapanema) (Fig. 2). Type 1 consists of a lenticular or dome-shaped deposit whose thickness and extension do not exceed 4 m and 80 m, respectively. Type 1 wet peperites were likely generated by thin lava flows (up to 5 m thick) which interacted with sediments that filled up ponds during previous flowing events. Type 2 wet peperites were formed by thicker lava flows (20–30 m) over sedimentary layers, resulting in peperite deposits whose thickness does not exceed 1.5 m. Speculative, our results suggest that Type 1 wet peperites exhibit a higher propensity for microfossil preservation compared to Type 2, with a greater abundance of microfossils.

## Results

### Biogenicity

The general appearance and preservation of the microfossils here studied distinguish them from potential abiogenic structure or pseudofossils^27–30^. Regarding biogenicity^27,28,31,32^, the organic walled microfossils meet all criteria that are commonly used, such as: (i) insertion of the fossil inside the rock, hampering later contamination; (ii) morphologically plausible size and shape; (iii) variation in preservational aspects; and (iv) presence of organic compounds.

### Taphonomy and diversity of organic walled microfossils

**Figure 3:**
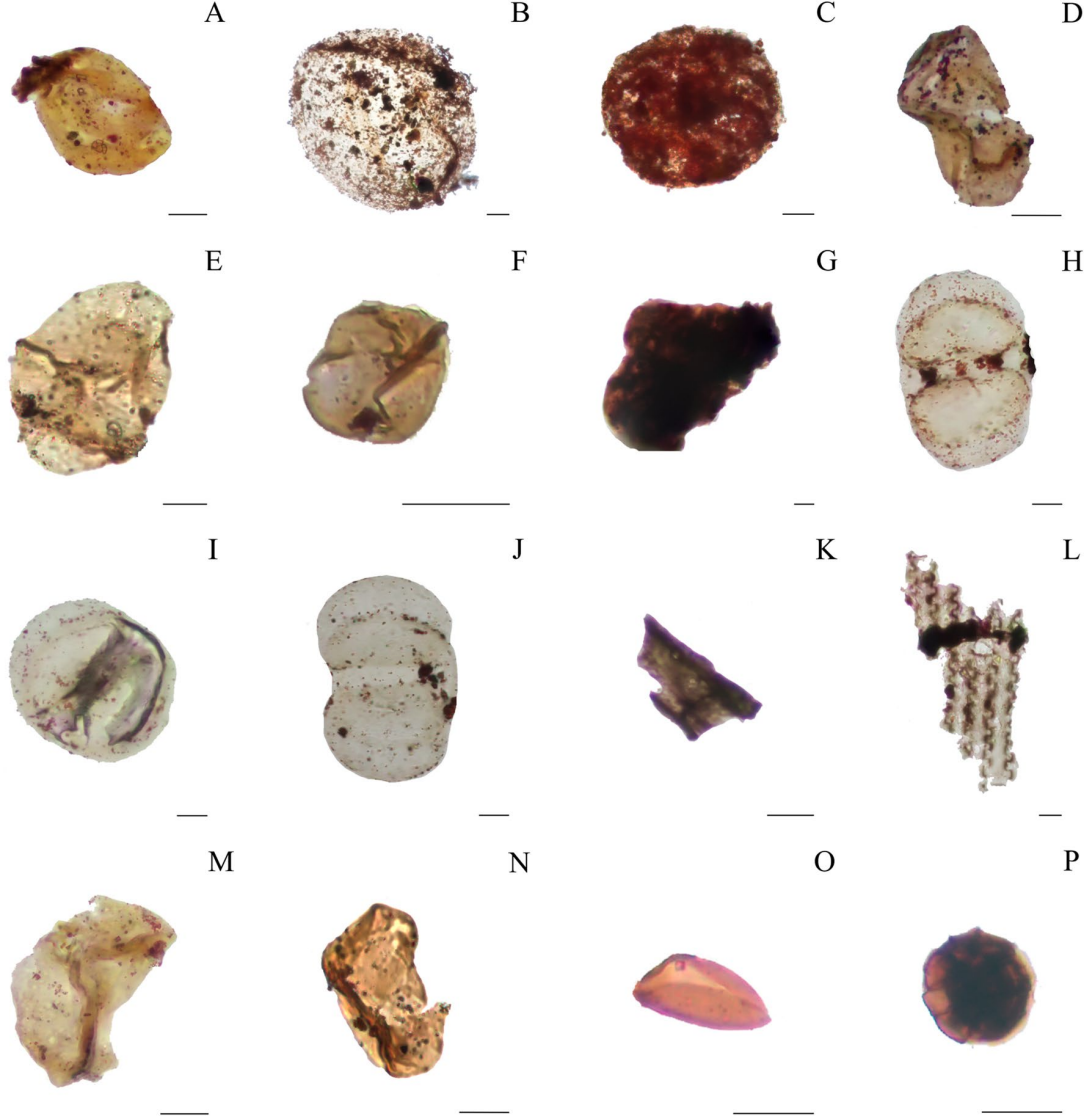
Organic walled microfossils from Paranapanema Formation wet-peperites. (**A**–**G**,**M**) palinomorphs and amorphous organic matter found at BS-22 site—*Inaperturopollenites* spp.; *Araucariacites* spp.; *Leiosphaeridia* spp.; *Callialasporites* spp.; *Araucariacites* spp.; *Inaperturopollenites* spp.; Amorphous Organic Matter and *Callialasporites* spp.. (**H**–**J**) *bisaccate* pollen grains found at BS-23 – *Dissacite* indeterminate. (**K**,**L**) well-preserved fragments of cuticle from PR 11. (**N**) *monosaccate?* pollen grain from PR 11—*Callialasporites* spp.. (**O**,**P**) fungi spores from PR-11—*Ascotaiwania* spp.. Scale: 20 μm.

We identified an assemblage (N = 71) of organic walled microfossils including pollen grains (N = 10, including two undifferentiated *bissacates)*, sporomorphs (N = 15, with some possible fern spores), spores (N = 1), acritarchs (N = 8), fungal spores (N = 5), phytoclasts (N = 15) and amorphous organic matter (N = 17; Fig. 3; Supplementary 4–6). The size of the organic walled microfossils varies from 11 μm to 208 μm. Terrestrial palynomorphs, including the pollen grains, are well-preserved, with color ranging from light yellowish to yellowish orange (low thermal alteration index (LTAI) = 1–2 + ; Fig. 3A,B,D–F,H–J and Supplementary 2–4)^28^. Although the preservation of the microfossils prevents identification of a precise affinity, there are no clear signs of carbonization/later diagenetic alteration. Fungal spores show an orange color with visible margin lines (Fig. 3O,P). Aquatic palynomorphs represented basically by acritarchs (Fig. 3C,G,M and supplementary 2–4) range from pale yellow to yellowish orange suggesting low degrees of thermal maturation. The majority of the phytoclasts, with color ranging from brown to gray, are identified as non-opaque (Fig. 3K,L and Supplementary 4–7).

### Taxonomic breakdown

Considering the expected taphonomic conditions related to the paleodepositional settings, particularly due to fine sand and volcanic thermal interaction, we have discovered organic-walled microfossils that can be associated with well-known Early Cretaceous families such as Araucariaceae (*Araucariacites* spp.; *Callialasporites* spp.) and Cupressaceae (*Callialasporites* spp.; Table 1 and Supplementary 9). In addition to various Gymnosperm specimens (Dissacate and Monossacate indeterminate), our findings also include acritarchs (*Leiosphaeridia* spp., *Quadrisporites* spp. and a few indeterminate species) along with Fungi representatives from Ascomycota, Conioschyphaceae type, as well as other unidentified hyphae and spores.

**Table 1:** Particulate organic matter and palynomorphs recorded from Serra Geral deposits.

Formation	Type of rock	Particulate organic matter/Palynomorphs	Affinity
Paranapanema	Wet peperites	*Araucariacites* spp.	Araucariaceae
		*Inaperturopollenites* spp.	Cupressaceae
		*Callialasporites* spp.	Araucariaceae/Podocarpaceae
		Dissacate indeterminate	Gymnosperm
		Sporomorph indeterminate	
		Dissacate indeterminate	Gymnosperm
		Monossacate indeterminate	Gymnosperm
		Pitted phytoclast	
		Leaf cuticle	
		Non-opaque phytoclast	
		Amorphous organic matter	
		*Leiosphaeridia* spp.	Acritarch
		*Quadrisporites* spp.	Acritarch
		Acritarch indeterminate	Acritarch
		Fungal hyphae	Fungi
		Fungal spore	Fungi
		*Ascotaiwania* spp?	Ascomycota
	Sandstones	Sporomorph indeterminate	
		Opaque phytoclast	
		Amorphous organic matter	
		Fungi	Fungi
Pitanga	Wet peperites	Sporomorph indeterminate	
		Non-opaque phytoclast	
		Amorphous organic matter	
		Fungal spore	Fungi
		Conioscyphaceae type	Ascomycota
	Interpillow sandstone	Sporomorph indeterminate	
		Non-opaque phytoclast	
Esmeralda	Wet peperites	Non-opaque phytoclast	
		Fungal spore	Fungi
Torres	Dry peperites	Amorphous organic matter	
		Fungal spore	Fungi
		Acritarch indeterminate?	Acritarch
		*Quadrisporites* spp.	Acritarch

### Composition and distribution

**Figure 4:**
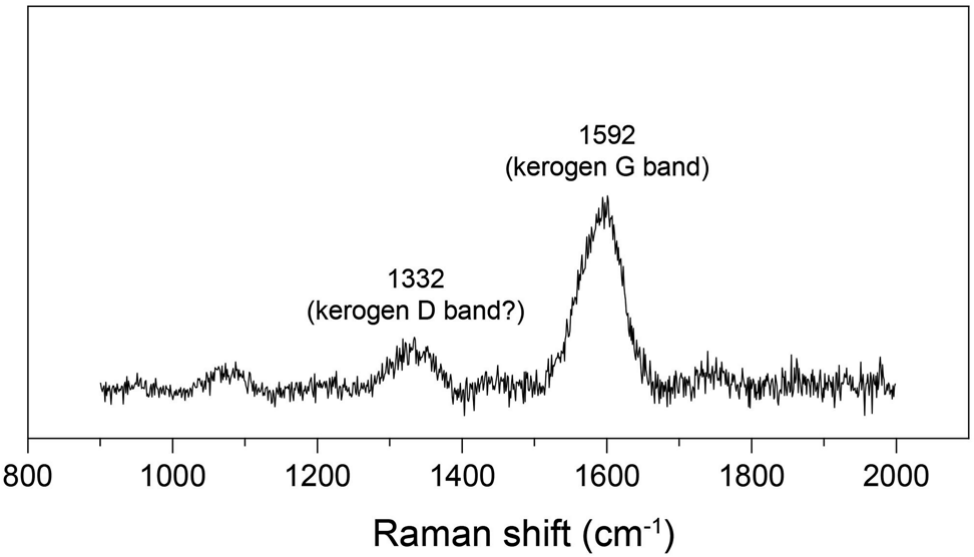
Raman spectrum of Vec-30 sporomorph from Torres Formation. The presence of kerogen is confirmed by the G band (at 1592 cm^−1^) and also possibly the D band (at 1332 cm^−1^).

Raman spectroscopy (Fig. 4) showed the typical kerogen bands in some of the studied microfossils: G band (ca. 1592 cm^−1^) and possibly the D band (ca. 1332 cm^−1^). Several palynomorphs seem to have inclusions or coverings of iron oxides that are identified as hematite through the typical Raman bands of this mineral (Supplementary 6). Due to this widespread presence of hematite, it is possible that there in some influence of the 2LO band of hematite at the region of the D band^30^. Nevertheless, the presence of the G band strongly supports the kerogenous composition of the analyzed fossils (Fig. 4).

**Figure 5:**
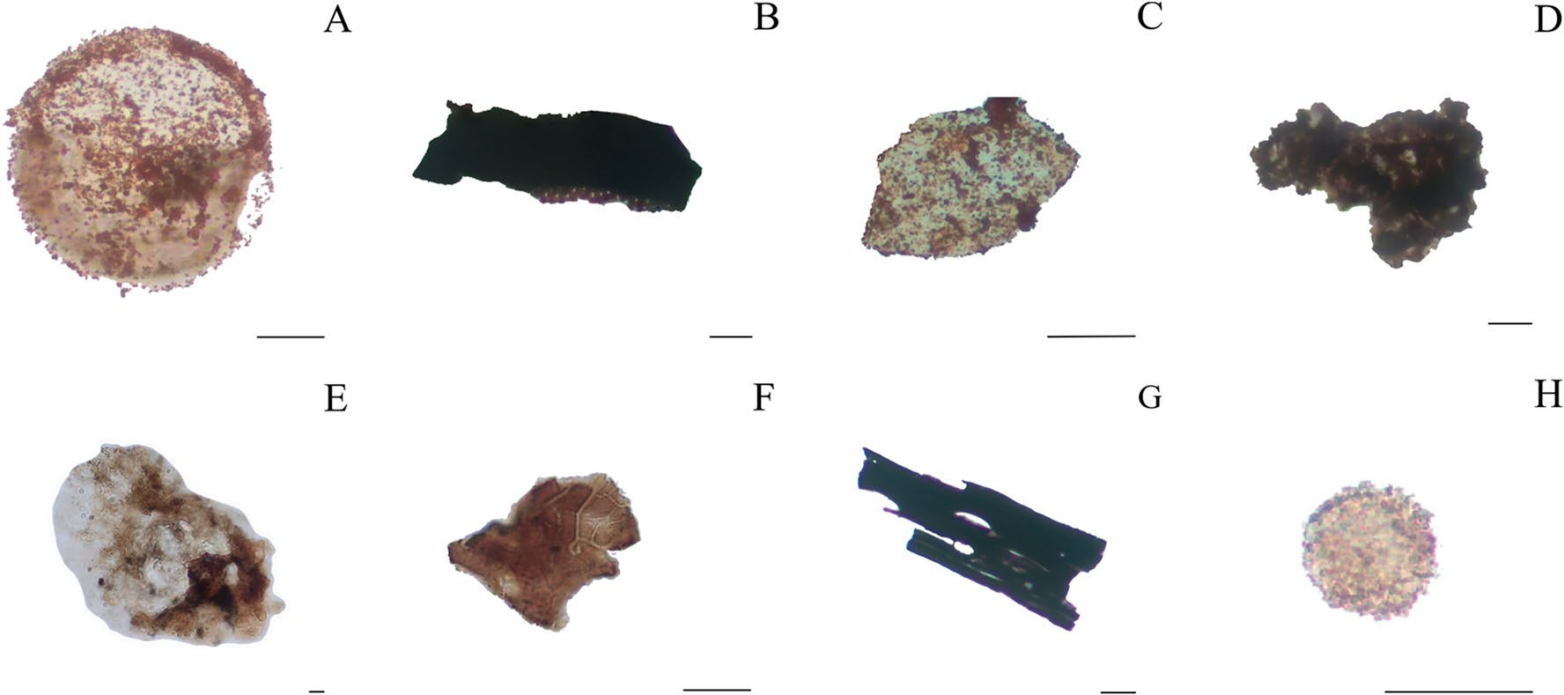
Preservation of organic walled microfossils in volcaniclastic deposits along Serra Geral Group. The varying preservation of organic walled microfossils within volcaniclastic deposits, showcasing distinct examples from different formations and stages of volcanic activity related to the Paraná-Etendeka LIP. (**A**,**B**) well-preserved palynomorph (*Leiosphaeridia* spp.) and pitted phytoclast, discovered in the later stages of volcanism, specifically in wet peperites of the Paranapanema and Pitanga formations. (**C**,**D**) rust palynomorph (*Baculatisporites* spp.?) and amorphous organic matter, recovered from "dry peperites" formed during the initial phases of volcanism in the Torres Formation. (**E**,**F**) pollen grain (*Araucariacites* spp.) and cuticle extracted from interpillow sandstones of the Pitanga Formation. (**G**,**H**) phytoclast and unidentified palynomorph (possibly a tardigrade egg?) encountered in heterolithic sandstones of the Paranapanema Formation. Scale: 20 μm.

Organic walled microfossils are variably distributed and preserved within the primary volcaniclastic deposits, with wet peperites showing higher abundance and better-preserved specimens than the heterolhitic sandstones, dry peperites and interpillow deposits (Fig. 5). Most of the 42 recovered microfossils from wet peperites have the general morphological ornamentation preserved, such as sacci delimitation, in the case of *bissacates* (Fig. 3A–N). The organic walled microfossils abundance and quality of preservation also seem to increase towards the upper formations (i.e., Torres Formation (N = 8); Pitanga Formation (N = 12) and Paranapanema Formation (N = 51)), which record the later stages of the volcanic event (Supplementary 7).

## Discussion

### Wet peperites as a new source of paleontological data in volcanic settings

Evidence of ancient life in the form of body and trace fossils is primarily known from sedimentary rocks, even though several studies have emphasized the existence of life in uncommon environments, such as the so-called deep biosphere (e.g., microorganisms found in hydrothermal vents, which are features associated with certain types of igneous activity)^33–40^. Indeed, previous reports have demonstrated that igneous and metamorphic rocks have the potential to preserve evidence of ancient life (microfossils and simply organic compounds) in unconventional geological settings beyond sedimentary rocks^33–40^.

Albeit rare, diverse types of fossils, ranging from vertebrates to plants, can be recovered from igneous deposits, although these are almost exclusively related to extrusive processes, such as ash and tephra falls, pyroclastic surges, lahars and pillow lavas^41–48^. Volcanic events even may have the potential for producing exceptional preservation, which can be attributed to rapid burial in extremely fine grained sediment^49^. For instance, fossils have been identified in volcanic/volcaniclastic deposits from the Ediacaran and throughout the Phanerozoic, with greater abundance and better preservation observed in more recent deposits^41–48^. Unlike ash and tephra falls that result in vertical sediment accumulation, lava flows and pyroclastic flows can be highly destructive, leading to limited fossil recovery in this context.

Although it is generally intuitively assumed that the high temperatures of molten lava would incinerate any organic material, thus destroying their potential as fossils, remains of large plants and animals can be preserved in rare instances, as evidenced by the tree molds in the July 1974 lava flow of Kilauea^48^ and the questionable preservation of a complete rhinoceros body within 15–16 Ma basalt flow in the Grand Coulee area^41^, Washington, USA. In the case of the preserved rhino, the lava flow was rapidly quenched by entering a subaqueous environment, such as a swamp, lake, or pond. Conversely, in the case of the lava trees in Hawaii, the slowing velocity and decreasing temperature of the flow were due to obstacles formed by previous flows. In both instances, biotic material was transported before deposition and burial.

In the context of our study, we hypothesize that a combination of transported material into an aqueous environment is responsible for the preservation of organic walled microfossils in PE-LIP. We find that the wet peperites contains a better preservation quality and higher abundance than the laterally correlated volcanoclastic heterolithic sandstones. Due to its similar sedimentary framework (fine sand with silt-mud laminae), one could expect the preservation potential to be similar in both settings. However, we propose that the greater preservation potential of fossils in wet peperites may be related to differences in their formation during early diagenesis, such as the interaction of flowing lava and wet sediments that would allow a more rapid cementation of the sediments.

### Paleoenvironmental significance and biotic indicators

**Figure 6:**
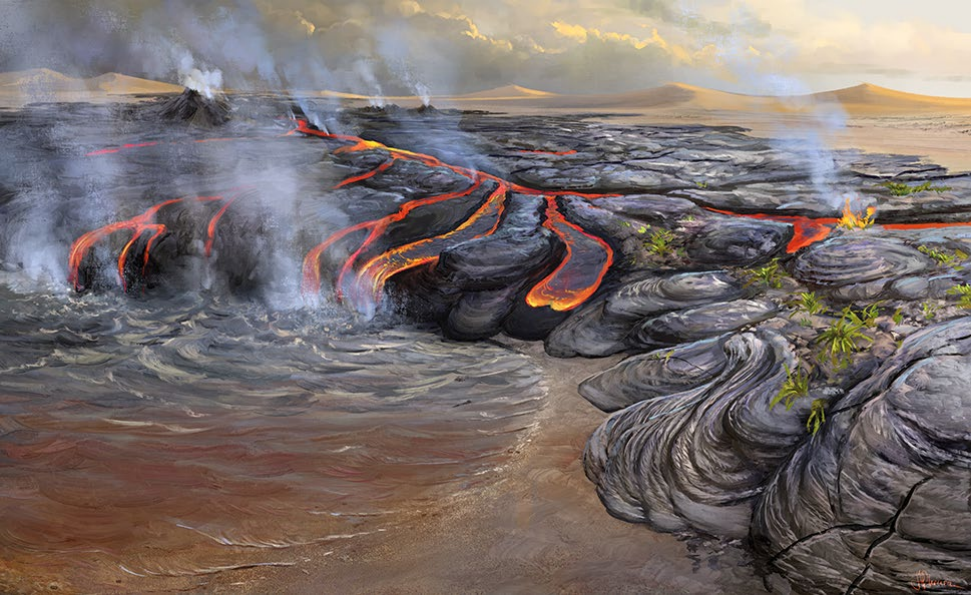
Paleoenvironmental Reconstruction of the Early Cretaceous Paraná Basin. The large volume eruptions produced profound change in the paleoenvironment and paleoclimate of the Parana Basin leading to suitable conditions to life. The volcaniclastic rocks allowed an understanding of the complex paleoenvironmental dynamics and the impact of volcanic events on the ancient ecosystem of the Paraná Basin. Artistic reconstruction by J. S. d’Oliveira.

The discovery of organic walled microfossils corroborate the former suggestion of subaqueous conditions in the intertrap deposits of PE-LIP, and support the interpretations for regional changes from desertic to more humid conditions during the Valanginian (139.4–133.9 Ma) in South-Central Gondwana^3–8^ (Figs. 5, 6). These changes may have been triggered by the emplacement of PE-LIP. The presence of organic microfossils identified in the studied sites, together with the geological context, suggest that these PE-LIP intertrap paleoenvironments may have been similar to endorheic ponds, especially in the upper formations (Pitanga and Paranapanema)^3–8,25^. The paleoclimate change during the later stages of the Parana volcanism is inferred based on a comprehensive analysis of sedimentary and volcaniclastic deposits. Initially, aeolian sedimentary deposits were found, indicating dry conditions consistent with the aeolian dunes in the Botucatu Formation^50,51^. However, further north in the province, there is a gradual transition towards more humid conditions. This shift is evidenced by sporadic occurrences of wet peperites in Rio Grande do Sul, followed by their increasing frequency in Parana state and ultimately the presence of pillow lavas in deposits from Sao Paulo and Minas Gerais's northernmost regions. Taken together, these observations suggest an escalating level of humidity as the volcanism progressed^6^.

Before the volcanism, the prevailing environmental conditions on South-Central Gondwana were warm and dry as evidenced by widespread aeolian sand dune deposits of the Botucatu Formation in Brazil and the coeval Twyfelfontein Formation in Namibia^1,20,51^. Aeolian dunes and dry peperite rocks interbedded with the early basalt flows in southernmost Brazil and Namibia^2,8,23,24,52^ suggest that these sand dunes systems remained active at the beginning of the volcanic outbreak. Although it has been suggested that hyper-arid conditions led to the establishment of dry peperites rocks throughout the Torres Formation^24^, more humid conditions could have been already present, since the microfossil and amorphous organic matter identified at the studied sites already indicates the presence of water, perhaps related to ephemeral ponds. Nevertheless, we cannot dismiss the possibility that the occurrence of palynomorphs reflects lava field morphology rather than climate change. It is known that organic fossils can be preserved in arid environments where vegetation tends to concentrate around certain areas. The volume of volcaniclastic and sedimentary rocks increases towards the upper sequences (Pitanga and Paranapanema formations), as well as the abundance of organic walled microfossils. The presence of *bissacates* and the potential existence of fern spores provide evidence suggesting more humid environmental conditions, possibly indicating these ferns as primary colonizers in previously barren lands. As ferns depend on water to germinate, they are generally associated with moist conditions and, consequently, rarely reported from arid environments^53^. Ferns are well-known as pioneering organisms that rapidly colonize and regenerate in the wake of catastrophic events, such as the Permian–Triassic boundary, Hawaii, and Krakatoa eruptions^49,54–57^. This resilience is attributed to their prolific spore production, their capacity to thrive in nutrient-poor substrates, and their ability for dispersal via wind, collectively highlighting their effective adaptation for colonizing and spreading to new regions^49^. In contrast, conifer pollen utilizes a different method for expanding its range by either originating from local vegetation or being carried long distances by the wind. Nevertheless, the presence of phytoclasts (opaque and non-opaque particles) and fungal spores suggests some proximity to the source area^58^, at least for some of the organic microfossils.

## Methods

### Material and analysis

Samples were collected from 20 outcrops throughout the Paraná Basin, 5 of them which were considered to be infertile for microfossils after the standard preparation. About 1.5 kg of rock from each site was prepared using the standard method^59^: all minerals were removed by hydrochloric and hydrofluoric acids. Material was sieved through a 10 μm mesh and mounted on permanent slides. This initial phase was done at Laboratório de Geoquímica II from Federal University of Santa Catarina, Brazil. These strew mounts were analyzed and photographed by using a camera mounted on a Zeiss Axio Imager A1 transmitted-light microscope at Laboratório de Micropaleontologia of the University of São Paulo, Brazil.

### Raman

Raman spectroscopy was performed at the Brazilian Research Unity of Astrobiology (NAP/Astrobio). We used a Renishaw inVia micro-Raman, with 532, 633, and 785 nm lasers. Maps were collected with a 100 × objective with step size of 0.7 µm.

### Supplementary Information

Supplementary Information.

## Data Availability

The rock samples, as well as the palynological and petrographic thin sections, are available to analysis at Institute of Geosciences, University of São Paulo (Brazil). We further provide a map detailing the area under investigation, but also additional microfossils pictures, Raman peaks and supplementary materials.
