# Supplementary material for: Organic walled microfossils in wet peperites from the early Cretaceous Paraná-Etendeka volcanism of Brazil
Source: Sci Rep. 2023 Sep 16;13:15362. doi: 10.1038/s41598-023-42483-6 (PMC10505181; doi:10.1038/s41598-023-42483-6)
Supplement: Supplementary file 1 — Supplementary Information. [file 41598_2023_42483_MOESM1_ESM.doc]

**Supplementary Material**

**Organic walled microfossils in wet peperites from the early Cretaceous Paraná-Etendeka volcanism of Brazil**

**Authors: Lucas Del Mouro^1,2*^, Bruno Becker-Kerber^2^; Valdecir A. Janasi^2^, Marcelo de Araújo Carvalho^3^, Breno L. Waichel^4^, Evandro F. Lima^5^, Lucas M. M. Rossetti^6^, Vinicius Cruz^7^; Mateus Souza Silva^4^; Natália Famelli^8^ & Javier Ortega-Hernández^1^**

1 Department of Organismic and Evolutionary Biology and Museum of Comparative Zoology, Harvard University, Cambridge, MA 02138, USA;2 Institute of Geosciences, University of São Paulo, São Paulo, Brazil; 3National Museum/Federal University of Rio de Janeiro, Brazil; 4Espepetro, Federal University of Santa Catarina, Florianópolis, Brazil; 5 Federal University of Rio Grande do Sul, Brazil; 6 Federal University of Mato Grosso, Brazil; 7CODES - Centre for Ore Deposits and Earth Sciences, University of Tasmania, 7001, Australia; 8 Centro de Pesquisas e Desenvolvimento Leopoldo Américo Miguez de Mello – CENPES/PETROBRAS, Brazil*e-mail:

*Corresponding author: e-mail: [ldmouro@usp.br](mailto:ldmouro@usp.br); ldelmouro@fas.harvad.edu

**Supplementary Text 1 – Evaluated paleodepositional settings**

The Paraná-Etendeka Large Igneous Province (PELIP) is a well-known continental flood basalts which was emplaced over an active dry aeolian system from Botucatu Desert^1-4^. The bulk of volcanism lasted approximately 3 Myr (134 – 131 Ma), however, most of the volcanism was supposed to be under 1 Myr. Along the stratigraphic pile, at least four different intertraps deposits are recognized.

In the first moments of the volcanism onset—Torres Formation—,the aeolian deposition related to Botucatu Desert was active as demonstrated by the dunes and dry peperites interbedded with the first basalt flows in southernmost igneous province ( Torres Formation)^1-5^. These dry peperites were supposed to be formed under no or minimum water conditions when the lava flow cascade down the front of unstable and unconsolitade dune faces (Fig. 1A). The putative presence of Amorphous Organic Matter (AOM) in the dry peperites indicates that water was present, once AOM is commonly derived from the degradation of phytoplankton or bacteria, in a process that requires water^6^.

Later in the volcanism pile— Pitanga and Paranapanema formations—, The conditions were humid as suggested by the presence of pillow lavas, lacustrine sediments (volcanoclastic heterolhitic sandstones) and wet peperites, predominantly in the northern Brazilian side of the province^7-8^. Pillow lavas can be formed by underwater eruption or by subaerial lava flow reaching subaqueous conditions. Therefore, the analyzed interpillow sandstones were generated after the interaction of pillow lavas with the fluvio-lacustrine sediments (sand-clay), which preserved the bedding and lamination features (Fig. 1B).

The wet peperites of PELIP are dominant to the later stages of the volcanism and mostly distributed along the Paraná State. Wet peperites-forming process occur in the border of the lake when the basaltic lava flows run into the water and mingling with the unconsolidated sediment (Fig. 1C). As seen in for the interpillow deposit, the bedding and lamination features were also preserved.

**Reference**

1. Scherer, C.M.S. 2000. Eolian dunes of the Botucatu Formation (Cretaceous) in southernmost Brazil: morphology and origin. Sedimentary Geology, 137, 63–84, https://doi.org/10.1016/S0037-0738(00)00135-4
2. Scherer, C.M.S. 2002. Preservation of aeolian genetic units by lava flows in the Lower Cretaceous of the Paraná Basin, Southern Brazil. Sedimentology, 49, 97–116,https://doi.org/10.1046/j.1365-3091.2002.00434.x
3. Jerram, D.A., Mountney, N.P., Howell, J.A., Long, D., Stollhofen, H., 2000. Death of a sand sea: an active aeolian erg systematically buried by the Etendeka flood basalts of NW Namibia. J. Geol. Soc. Lond. 157, 513–516. https://doi.org/10.1144/jgs.157.3.513.
4. Jerram, D.A., Stollhofen, H., 2002. Lava/sediment interaction in desert settings: are all peperite-like textures the result of magma–water interaction? In: Skilling, I.P., White, J.D.L., McPhie, J. (Eds.), Peperite: Processes and products of magma–Sediment Mingling. J. Volcanol. Geotherm. Res., vol. 114, pp. 231–249.
5. Waichel, B.L., Scherer, C.M.S., Frank, H.T., 2008. Basaltic lava flows covering active aeolian dunes in the Paraná Basin in southern Brazil: Features and emplacement aspects. J. Volcanol. Geotherm. Res. 171, 59–72.
6. Tyson, R.V., 1995. Sedimentary Organic Matter. Organic Facies and Palynofacies. Chapman and Hall, London, 615.
7. Waichel, B.L., Lima, E.F., Sommer, C.A., Lubachesky, R., 2007. Peperite formed by lava flows over sediments: an example from the Central Paraná Continental Flood Basalts, Brazil. J. Volcanol. Geotherm. Res. 159, 343–354. https://doi.org/10.1016/j.jvolgeores.2006.07.009.
8. De Moares, L. C., Seer, J.H., 2018. Pillow lavas and fluvio-lacustrine deposits in the northeast of Paraná Continental Magmatic Province, Brazil, Journal of Volcanology and Geothermal Research, v. 355,78-86. https://doi.org/10.1016/j.jvolgeores.2017.03.024

**Supplementary Figure 1. Paleodepositional settings during the Paraná-Etendeka volcanism.** (A) Dry peperite-forming process with almost no water in the beginning of volcanism (Torres Formation). (B) Interpillow sandstone from Pitanga Formation. (C) Wet peperite-forming process in later stages of the volcanism (Pitanga and Paranapanema formations). Model drawn using Corel Draw x8.

**Supplementary Figure 2.** Each studied outcrop in the Serra Geral Group, Paraná-Etendeka, where we recovered organic walled microfossils: Dry peperites: 1 - VN26 **(a)**; 2 - VEC30A **(b)**; 3 - VEC12 **(c)**; Dacite: 4 - VN12 **(d)**; Interpillow sandtones: 5 - NFUB03**(e)**; 6 - UBNF32A **(f)**; Wet Peperites: 8 - PR06 **(g)**; 9 - PR11 **(h)**; Heterolithic sandstone: 1 - PR02 **(i)**; Wet peperites: 11 - BS18 **(j)**; 12 - BS12 **(k)**; 13 - BS23 **(l)**; 14 - SC03/BS01 **(m)**; 15 - BS22 **(n)**; 16 - BS24 **(o)**; 17 - BS21 **(p)**.

**Supplementary Figure 3.** Rock samples. **(a-d)** Dry peperites, wet peperites, and heterolithic sandstone samples. **(e-g)** The remaining residue after chemical and physical preparation**.(h-j)** Petrographic thin sections were obtained from the study sites**.(h-i)** Interpillow sandstones **(**NFUB03 and UBNF32A**)** with potential ostracod fragments.**(j)** A thin section of wet peperite (BS22) with a potential microfossil preservation **(k)**.

**Supplementary Figure 4.Additional Organic walled microfossils from Paranapanema Fm.** (a) unidentified phytoclast?. (b, n) I pollen grain. (c,m) sporomorphs. (d, j, l) acritarch?. (e, f, h, k, o) unidentified biogenic clast. (g) monosaccate pollen grain. (i) inaperturate pollen grain. Scale: 20 μm.

**Supplementary Figure 5. Additional OW microfossils from Paranapanema Formation, part 2.** (a-e) palynomorphs found at SC-03. (f-s) owm recovered from volcaniclastic sandstones PR-02. (f-g, j-n) palynomorphs. (h-i) amorphous organic matter; unidentified biogenic clasts; (p-t) microfossils from the PR-11 peperite; (p, s, t) non-opaque phytoclast; (q-r) sporomorphs *monosaccate* pollen grain. Scale: 20 μm

.

**Supplementary Figure 6. Organic walled microfossils from Pitanga and Torres Formation.** (a-i) Pitanga formation microfossils; (a-d) palynomorphs from NF_UB_03 interpillow; (a,d) sporomorph; (b) non-opaque phytoclast; (c) amorphous organic matter; (e-i) palynomorphs recovered from PR-06 wet peperite: ((g) amorphous organic matter; (h) sporomorph; (I, k, l) non-opaque phytoclasts; (j) fungal spore. Scale: 20 μm.

**Supplementary Figure 7**. **Raman spectrum from the hematite coatings/inclusions of the volcanic carbonaceous microfossils.** The majority of specimens analyzed through Raman spectroscopy shows a spectrum with peak at1323 cm-1, which is related to hematite.

**Supplementary Figure 8**. **A flowchart of the traditional acid-based palynological preparation technique adapted to Mesozoic volcanoclastic samples.** The majority of the process is commonly known and applied, however, successful recovery of microfossils will required at least 1.5 kg of crushed rocks.

**Supplementary 9**

| Outcrop | Formation | Location(utm) | Altitude | Type of Rock | Figure | Description | Possible Affiliation | Regional State |
| --- | --- | --- | --- | --- | --- | --- | --- | --- |
| **BS 22** | Paranapanema | 249645x704519 | 657 m | Wet peperite | Fig. 3 - A | Inaperturate pollen grain with a circular to semi-circular shaped and size of 40.23 smooth. | Inaperturopollenites spp.. ? | **Santa Catarina** |
|  |  |  |  |  | Fig. 3 - B | oval-spherical sporomorph strongly affected by diagenesis. Diameter of 208.85 μm and exine at least with psilate surface. Below the coarse fragments, a pale sulcate mark can be observed. Thereby, this sporomorph resembles monosulcate pollen grains. | *Araucariacites* spp.? |  |
|  |  |  |  |  | Fig. 3 - C | Oval shaped sporomorph of 44.17 μm. The exine is smooth. | Fresh water Acritarch. Possible *Leiosphaeridia* spp |  |
|  |  |  |  |  | Fig. 3 - D | Partially preserved bisaccate pollen grain with 73.25 μm. A hollow aberture in the central of the sporomorph and a delimited sacci on the left. The specimen is affected by the diagenesis conditions. | Bissacate. Callialasporites spp. |  |
|  |  |  |  |  | Fig. 3 - E | Bisaccate pollen grain with Y-shaped mark. The two sacci in equatorial position and the exine is smooth. Size of 52.1 μm. | *Araucariacites* spp. or *Inaperturopollenites* spp. ? |  |
|  |  |  |  |  | Fig. 3 F | Inaperturate pollen grain with a circular to semi-circular shaped and size of 27.21 smooth. | Inaperturopollenites spp.. ? |  |
|  |  |  |  |  | Fig. 3 G | Amorphous Organic Matter | Amorphous Organic Matter |  |
|  |  |  |  |  | Suppl. 2A | Regular broken phytoclast (39.94 μm) | Possible cuticle |  |
|  |  |  |  |  | Suppl. - 2B | Lateral view of a bisaccate pollen grain. The two sacci are not well delimited and is folded. The exine is smooth and shaped mark is indistinct. | Dissacate indeterminate |  |
|  |  |  |  |  | Suppl. - 2C | Small conical shape sporomorph with diameter of 29.41 μm. Margins show minor wrinkles, possible "psilate" type. | sporomorph |  |
|  |  |  |  |  | Suppl. - 2D | A spherical shape palynomorph with diameter of 94.75 μm and a smooth vesicle wall. The acritarc has sufferred a intense diagenetic process and it is possible to observed a well-delimited ring. | Acritarch. *Leiosphaeridia* spp.? |  |
|  |  |  |  |  | Suppl. - 2E | Oval shaped element with 34.73 μm. | indeterminate |  |
|  |  |  |  |  | Suppl. -2F | Small rounded shape element with 26.56 μm of diameter. | indeterminate. |  |
|  |  |  |  |  | Suppl. 2G | Monosaccate pollen in lateral viiew. Amb circular to sub-circular. Size of 72.58 μm. | Callialasporites spp.? |  |
|  |  |  |  |  | –Fig 3. M | Broken monosaccate pollen in lateral 15iew. Amb circular to sub-circular. Blurry triradiate scar, exine is smooth. | Callialasporites spp.? |  |
|  |  |  |  |  | Suppl. - 2H | Unidentified element with smooth margins and triangular shape (94.42 μm) | Spore indeterminate. |  |
|  |  |  |  |  | Suppl. - 2I | Almost completed inaperturate pollen grain with a semi-circular shaped and size of 35.88 μm. The exine is not entire preserved and shows psilate surface. | Inaperturopollenites spp. ? |  |
|  |  |  |  |  | Suppl. - 2J | A spherical shape palynomorph with diameter of 85.65 μm and a smooth vesicle wall. The acritarc has sufferred a intense diagenetic process and it is possible to observed a well-delimited ring. | Acritarch? *Leiosphaeridia* spp.? |  |
|  |  |  |  |  | Suppl. 2K | Amorphous Organic Matter | Amorphous Organic Matter |  |
|  |  |  |  |  | Suppl. 2M | Small altered sporomorph (26.62 μm) with round shape and minors process on the margin. | Fungal spore. |  |
|  |  |  |  |  | –Fig, 4A | Spherical shape palynomorph with diameter of 77.55 μm. The acritarc has sufferred a intense diagenetic alteration, however the vesicle wall seems to be "psilate". | Fresh water Acritarch. Possible *Leiosphaeridia* spp |  |
|  |  |  |  |  | Suppl. - 2L | Spherical shape palynomorph with diameter of 59.12 μm. The acritarc has sufferred a intense diagenetic alteration, however the vesicle wall seems to be "psilate". | Fresh water Acritarch. Possible *Leiosphaeridia* spp |  |
|  |  |  |  |  | Suppl. - 2N | Bissacate pollen grain in dorsal view. The two sacci are well defined and with possible reticulate sculpture. The polle grain with 59.41 μm of diameter and well affected by the diagenesis. | Dissacate indeterminate |  |
|  |  |  |  |  | Suppl. – 2O | Fungi. | indeterminate |  |
|  |  |  |  |  | Suppl. 2P | Rounded fungal spore of 25.15 μm. | Fungal spore indeterminate. |  |
|  |  |  |  |  | Suppl. 2Q | Coarsed rounded Acritarch with 48.59 μm. | Acritarch. *Quadrisporites spp.?* |  |
| **SC-03** | Paranapanema | 340012x7013162 | 690 | wet peperite | Suppl. 3. - C | Amorphous or degraded acritarch. fragment of 12.63 μm. | Acritarch? |  |
|  |  |  |  |  | – Suppl. 3D | A small circular shaped palynomorph ( 17.45 μm) with possible rugulate sculpture. | Acritarch indeterminate. |  |
|  |  |  |  |  | Suppl. 3. - F | A circular shaped sporomorph with 27.67 μm of diameter and sculpture rugulate. | Sporomorph indeterminate |  |
|  |  |  |  |  | Suppl. 3. - E | Circular to oval-shaped sporomorph with smooth or psilate surface. Specimen has 40 μm of diameter and preservation blurried aditional taxonomic features. | Sporomorph indeterminate |  |
|  |  |  |  |  | Suppl. 3. - G | Spherical shape palynomorph with diameter of 53.19 μm, preservation conditions masked further features. May be an acritarch. Probably initially affected by bacterial decomposition (aOM) | Sporomorph or Fresh water Acritarch (Quadrisporites spp ?) |  |
| **BS 23** | Paranapanema | 249679x7058087 | 720 | Wet peperite | Fig 3. - I | Compressed Bissacate pollen grain in dorsal view. The two sacci are delimited. The polle grain with 45.41 μm of diameter and well affected by the diagenesis. | Dissacate indeterminate |  |
|  |  |  |  |  | Suppl. 3. - A | Phytoclast and a palynomorph fragment and under amorphizationn process | AOM? |  |
|  |  |  |  |  | Suppl. 3. - B | Unidentified palynomorph with signs of amorfization. Diameter of 36.7 μm , margins indefined but resemble a fungi spore. | Fungi spore? |  |
|  |  |  |  |  | Fig 3. - H | Well defined bissacate pollen grain in dorsal view. The two sacci are well defined and with possible reticulate sculpture. The polle grain with 68.31 μm of diameter and well affected by the diagenesis. | Dissacate indeterminate |  |
|  |  |  |  |  | Fig 3. J | Well defined bissacate pollen grain in dorsal view. The two sacci are well defined and with possible reticulate sculpture. The polle grain with 156.80 μm of diameter and well affected by the diagenesis. | Dissacate indeterminate |  |
| **BS 12** | Paranapanema | 333249x6984163 | 455 | Volcanoclastic Sandstones | Suppl. 3. - I | Amorphous Organic Matter | Amorphous Organic Matter |  |
|  |  |  |  |  | Suppl. 2. - H | Amorphous Organic Matter | Amorphous Organic Matter |  |
| **BS 18** | Paranapanema | 299146x7002982 | 300 | Wet peperite | –Not Illustrated | Kerogenized spore with 32.38 μm. | spore |  |
| **PR 02** | Paranapanema | 602113x743877 | 597 | HeterolhiticSandstone | Suppl. 3J | A small circular shaped sporomorph with 16.57 μm of diameter and scabrate surface | sporomorph | **Paraná** |
|  |  |  |  |  | Suppl. 3K | Broken indeterminate biogenic structure. Sie of 60.87 μm in diameter. | Indeterminate. |  |
|  |  |  |  |  | Suppl. 3L | Small sporomorph (23.66 μm) with thick envelope and inclusions. Surface is smooth. | sporomorph |  |
|  |  |  |  |  | –Fig. 4G | Opaque lath with 137.26 μm long. | phytoclast - opaque lath |  |
|  |  |  |  |  | –Fig. 4H | A small circular shaped sporomorph with 18.02 μm of diameter and scabrate surface | sporomorph |  |
|  |  |  |  |  | Suppl. 3I. - | Amorphous organic matter. | AOM |  |
|  |  |  |  |  | Suppl. 3.M | Rounded shaped indeterminate biogenic structure. | Indeterminate |  |
|  |  |  |  |  | Suppl. 2. - Q | Fungal spore. | fungal spore |  |
|  |  |  |  |  | Suppl. 3. N | Indeterminate kerogenized oval-shape specimen. Maximμm length of 47.09 μm. Resembles fungal spore | Fungal spore?. |  |
|  |  |  |  |  | Suppl. 3H | Amorphous Organic Matter. Size of 34.23 μm. | AOM |  |
| **PR 11** | Paranapanema/Pitanga | 539189x7437185 |  | Wet peperite | Fig 3. - L | Well preserved cuticle. Size of 100.82 μm | phytoclast - non-opaque - Cuticle |  |
|  |  |  |  |  | Not Illustrated | Non-opaque phytoclast. Size: 60.69 μm long. | phytoclast - non-opaque |  |
|  |  |  |  |  | Not Illustrated | Pitted phytoclast. Size of 67.58 μm | phytoclast - non-opaque |  |
|  |  |  |  |  | Fig 3. K | Fragment of cuticle with 44.07 μm of length. | phytoclast - non-opaque - Cuticle |  |
|  |  |  |  |  | Fig 3. N | Partially preserved bisaccate pollen grain with 74.15 μm. A hollow aberture in the central of the sporomorph and a delimited sacci on the left. | *Callialasporites* spp.? |  |
|  |  |  |  |  | Suppl. 2. - T | Degraded Non-opaque phytoclast. Size: 23.53 μm long. | phytoclast - non-opaque |  |
|  |  |  |  |  | Suppl. 3Q. | Indeterminate sporomorph. Semi-circular shape with possible lines at center and smooth margin. Preservation conditions masked taxonomic features. Size: 20 μm of diameter. | Fungal spore? |  |
|  |  |  |  |  | Suppl. 3O | Blurred rounded sporomorph. Surface walls seems to be smooth and inside marks is present. Size: 18 μm. | Sporomorph - Fungal spore? |  |
|  |  |  |  |  | Suppl. 3R. | Circular palynomorph with fine isolate processes. Acritarch? Diameter of 32.72 μm. | Palynomorph – Acrirtarch or Fungal spore? |  |
|  |  |  |  |  | Suppl. 3S | Broken non-opaque phytoclast. Size of 19.82 μm | phytoclast - non-opaque |  |
|  |  |  |  |  | Suppl. 3T | Degraded non-opaque phytoclast. Size of 44.72 μm | phytoclast - non-opaque |  |
|  |  |  |  |  | –not illustrated | Small spherical palynomorph (11.82 μm of diameter). Exine probably psilate. | sporomorph |  |
| **PR 06** | Pitanga | 569906x7423357 |  | Wet peperite | Suppl. 4E | Amorphous organic matter. Size: 54.88 μm long. | AOM |  |
|  |  |  |  |  | Suppl. 4I | Spherical biogenic structure, possible a sporomorph under amorphization process. with 42.67 μm long. Despite the preservational conditions, the exine seems to be smooth and inclusions can be observed. The central cavity is thicker. | Indeterminate sporomorph. |  |
|  |  |  |  |  | Fig. 4B | Pitted phytoclast. Size: 133.31 μm long. | phytoclast - non-opaque - pitted |  |
|  |  |  |  |  | Suppl. 4F | Circular fungal spore. Size: 50.11 μm in diameter | fungal spore |  |
|  |  |  |  |  | Suppl. 4G - | Degraded non-opaquephytoclast. It seems to be under amorphization process. Size: 48.08 μm long. | Phytoclast - non-opaque |  |
|  |  |  |  |  | Suppl. 4H | Degrade non-opaque phytoclast. Size: 50.52 μm long. | phytoclast - non-opaque |  |
| **NF-UB-03** | Pitanga | 792609x7926903 |  | Interpillow- sandstone | –fig. 4E | Inderteminated sporomorph. Oval construction, 324 μm long. In upper section, fine limit conection given a impression of sacci delimitation. | probably Inaperturopollenites spp. or Araucaricites spp.? | **Minas Gerais** |
|  |  |  |  |  | Suppl. 4C | Big non opaque phytoclast, which was probably under the amorphization process. | phytoclast - non-opaque |  |
|  |  |  |  |  | Suppl. 4A | Rounded shape specimen with irregular surface. Preservation oblitared taxonomic features, but, it resembles spore. | sporomorph |  |
|  |  |  |  |  | Suppl. 4E | Rounded shape sporomorph with diameter of 52.13 μm. The exine seems to be psilate and the surface may be scabrate. | Sporomorph |  |
|  |  |  |  |  | Fig. 4 F | Degraded cuticle with 49.94 μm long | phytoclast - non-opaque - Cuticle |  |
| **UB-NF-32A** | Pitanga | 792768x7926923 |  | Interpillow- sandstone | Suppl. 4B | Unidentified biogenic material. Resemble non-opaque phytoclast affected by heat. Size: 32.35 μm | unidentified. |  |
| **VN 12** | Esmeralda (Cx. Dacites) |  | 300 | Wet peperite | Suppl. 4J | Degraded cuticle. Size: 131.26 μm long | phytoclast - non-opaque | **Rio Grande do Sul** |
|  |  |  |  |  | Suppl. 4K | Indeterminate biogenic particle.. Resemble a fragment related to sporomorph. Size: 36.67 μm | unidentified. |  |
| **VEC 12** | Torres |  | 128 | Dry peperite | Suppl. –4L | Acritarch degraded and under amorphization process. Size: 27.32 μm long. | Probably a *Quadrisporites spp.?* |  |
|  |  |  |  |  | Fig, 4C | Amorphized palynomorph. Size: 50.81 μm long. | Palynomorph – *Baculatisporites* ssp.? |  |
| **VEC 30A** | Torres | 340865x6733122 | 367 | Dry peperite | Suppl. 4M | Round small sporomorph ( 26.68 μm diameter). Surface seems to be corrugate. Probably a fungal spore. | Fungal spore? |  |
|  |  |  |  |  | Suppl. –4N | Pseudoamorphous phytoclast. Size: 135.56 μm long. | AOM |  |
| **VN 26** | Torres | 446438x6789950 | 160 | Dry peperite | Suppl. 4O | Amorphous organic matter | AOM |  |
|  |  |  |  |  | –fig. 4D | Amorphous organic matter | AOM |  |

**Table 1. Descriptive table regarding microfossils found along the Paraná-Etendeka system at Paraná Basin.**
